# Estimating the risk of rabies transmission to humans in the U.S.: a delphi analysis

**DOI:** 10.1186/1471-2458-10-278

**Published:** 2010-05-26

**Authors:** Sagar A Vaidya, Susan E Manning, Praveen Dhankhar, Martin I Meltzer, Charles Rupprecht, Harry F Hull, Daniel B Fishbein

**Affiliations:** 1 Combined Internal Medicine-Pediatrics Program, Mount Sinai School of Medicine, 1 Gustave Levy Place, New York, NY 10128 USA; 2 Preventive Medicine Residency, Career Development Division, Office of Workforce and Career Development, Centers for Disease Control and Prevention, 1600 Clifton Road NE, Atlanta, GA 30333 USA; 3 Immunization Services Division, National Center for Immunizations and Respiratory Diseases, Centers for Disease Control and Prevention, 1600 Clifton Road NE, Atlanta, GA 30333 USA; 4 Division of Emerging Infections and Surveillance Services, Coordinating Center for Infectious Diseases, Centers for Disease Control and Prevention, 1600 Clifton Road NE, Atlanta, GA 30333 USA; 5 Division of Viral and Rickettsial Diseases, National Center for Zoonotic, Vector-Borne and Enteric Diseases, Centers for Disease Control and Prevention, 1600 Clifton Road NE, Atlanta, GA 30333 USA; 6 Minnesota Department of Health, P.O. Box 64975, St. Paul, MN 55164 USA; 7 H. F. Hull & Associates, LLC, 1140 St. Dennis Court, St. Paul, MN 55116 USA; 8 Division of Global Migration and Quarantine, National Center for Preparedness, Detection, & Control of Infectious Diseases, Centers for Disease Control and Prevention, 1600 Clifton Road NE, Atlanta, GA 30333 USA

## Abstract

**Background:**

In the United States, the risk of rabies transmission to humans in most situations of possible exposure is unknown. Controlled studies on rabies are clearly not possible. Thus, the limited data on risk has led to the frequent administration of rabies post-exposure prophylaxis (PEP), often in inappropriate circumstances.

**Methods:**

We used the Delphi method to obtain an expert group consensus estimate of the risk of rabies transmission to humans in seven scenarios of potential rabies exposure. We also surveyed and discussed the merits of recommending rabies PEP for each scenario.

**Results:**

The median risk of rabies transmission without rabies PEP for a bite exposure by a skunk, bat, cat, and dog was estimated to be 0.05, 0.001, 0.001, and 0.00001, respectively. Rabies PEP was unanimously recommended in these scenarios. However, rabies PEP was overwhelmingly not recommended for non-bite exposures (e.g. dog licking hand but unavailable for subsequent testing), estimated to have less than 1 in 1,000,000 (0.000001) risk of transmission.

**Conclusions:**

Our results suggest that there are many common situations in which the risk of rabies transmission is so low that rabies PEP should not be recommended. These risk estimates also provide a key parameter for cost-effective models of human rabies prevention and can be used to educate health professionals about situation-specific administration of rabies PEP.

## Background

Approximately 6,000 to 7,000 cases of animal rabies are reported each year to the CDC, while there were 19 human cases indigenously acquired over the period 2000 - 2006 [1]. Annually, 16,000-39,000 people in the United States are potentially exposed to rabies and receive post-exposure prophylaxis (PEP), and none has ever developed rabies [2]. However, it is unclear whether the rarity of human rabies in the United States is due to low risk of acquisition, the effectiveness of rabies PEP, or both [3].

The number of rabid domestic animals (the most common vector of human rabies in the 20^th ^century in the United States as well as most other parts of the world) has also decreased. The number of PEP courses -- the direct cost of which is approximately $2,500 per fully treated patient -- has increased without any decrease in the number of human rabies cases [2,4,5]. Thus, PEP is often administered under inappropriate circumstances [6,7]. Health practitioners lack information allowing them to estimate the risk of rabies, complicating decisions on how to proceed when dealing with various low-risk rabies encounters, such as non-bites [8].

Currently, little information exists on the risk of rabies transmission in situations in which the risk is low, and controlled studies in humans are neither feasible nor ethical. Therefore, we used the Delphi technique to estimate (1) the risk of rabies transmission to humans and (2) the circumstances following which rabies PEP should not be recommended in various potential exposure scenarios common in the United States.

## Methods

This study was classified as exempt from human subject regulations by the Institutional Review Board of the Centers for Disease Control and Prevention. Recent studies have shown the utility of using the Delphi technique to obtain information about vaccines in situations where clinical trials are not feasible [9]. We developed a two-part protocol to estimate (1) the risk of rabies transmission to humans and (2) the circumstances following which rabies PEP should/should not be recommended. We used the Delphi technique to obtain a group consensus estimate of the risk of human rabies infection and the treatment practices using various potential exposure scenarios common in the United States [10]. The group was made up of public health professionals with experience in making decisions regarding administration of human rabies PEP on a regular occupational basis. A list of the panel members is provided in the Acknowledgment section.

Potential participants received an introductory questionnaire by email that requested information regarding the duration and type of experience they have had in recommending rabies PEP. The email clearly indicated that participation in the study was purely voluntary. After selection of the study panel, the study coordinator (S.A.V.) distributed a questionnaire consisting of seven scenarios of potential rabies exposures to different species (see Table 1, Appendix 1). In six of the scenarios, the animal responsible was not available for rabies testing. Three of the exposures were unprovoked bites and three were unprovoked licks by animals. The seventh was a possible exposure to a human rabies patient. Each scenario provided an estimate of the prevalence of rabies in the involved animal species based on information from published literature as follows: skunk 25%, bat 15%, cat 1%, and dog 0.1% [3,11,12]. Participants were also provided reference values of actual rabies mortality following different exposures to proven rabid animals based on the following published values: superficial bite to the hand: 5%; contact with rabid saliva on a recent wound: 0.1%; contact with rabid saliva on a wound older than 24 hours: 0.0% [13]. Finally, all scenarios standardized the post-exposure course with "the wound is not washed or cleaned" and "the animal cannot be found" in order to maintain clarity and consistency across potential exposure scenarios.

**Table 1:** Scenarios presented in the Delphi Questionnaire^¶.^.

Scenario	Animal	Contact scenario	Prevalence*
1	Skunk	Bite	25%

2	Bat	Unknown**	15%

3	Dog	Bite	0.1%

4	Dog	Lick	0.1%

5	Cat	Bite	1%

6	Cat	Lick	1%

7	Human	Unknown***	100%****

¶ See Appendix for complete questionnaire containing full descriptions of each scenario.

* Estimated prevalence rate consistent with published rates of animal rabies

** No obvious bites or skin abrasions, however bat bites are often not visible and patient history was unreliable (infant)

*** No bites, scratches, or direct contact with patient's saliva, however close contact for ten days (nursing)

**** The human exposure was a confirmed rabies case

In each round of the Delphi survey, responses from the participants were recorded anonymously under a pseudonym. For subsequent rounds, each participant received the aggregate results of the previous round and was asked to complete the questionnaire again taking into account the results from the previous round. The participants were also encouraged to provide comments regarding why they agreed or disagreed with the aggregated results and to explain the thought process behind their estimates. Relevant comments were anonymously included in subsequent rounds, with the anonymity of all responses preserved by editing explanations so that the respondent could not be identified. The questionnaire was completed by all the participants in three sequential rounds, by which time the results had stabilized and respondents were firm in their responses. The estimates for the risk of rabies transmission and the number of respondents recommending rabies PEP were calculated for each scenario and for all scenarios combined.

## Results

We contacted 27 public health professionals with significant experience in the fields of rabies and PEP. Twenty participants completed the study, four did not reply to the initial survey, two declined due to insufficient time, and one was unable to complete the final round due to medical illness. All participants had experience in assessing risk of rabies transmission and in recommending rabies PEP ranging from 2 years to over 30 years (median = 13) and 75 to >6000 individual rabies consultations (median = 1700). Most (75%) participants were affiliated with state public health departments while 25% were affiliated with national public health departments.

A summary of the panel's estimates for risk of rabies can be found in Table 2. The panel rated the first scenario, which described a bite from a skunk in an area in which 25% of tested skunks were positive for rabies, as representing the greatest risk of human rabies infection. Only 4 participants provided estimates different than the median value of 0.05. All participants indicated that they would recommend rabies PEP in such a scenario.

**Table 2:** Estimates of the Risk of Rabies Transmission^a^.

Scenario	**Exposure **^**b**^	**25**^**th **^**percentile**	**50**^**th **^**percentile**	**75**^**th **^**percentile**	Range	**Recommend PEP (% "yes")**^**c**^
**1**	Skunk (bite)	0.05	0.05	0.05	0.1 - 0.01	100

**2**	Bat (unk)	0.0001	0.001	0.001	0.01 - 0.000001	100

**3**	Dog (bite)	0.00001	0.00001	0.0001	0.001 - 0.00001	95

**4**	Dog (lick)	0.000001	0.000001	0.000001	0.00001 - 0.000001	10

**5**	Cat (bite)	0.0001	0.001	0.001	0.01 - 0.00001	100

**6**	Cat (lick)	0.000001	0.000001	0.000001	0.0001 - 0.000001	5

**7**	Human (unk)	0.000001	0.000001	0.000001	0.00001 -0.000001	20

^**a **^Study participants were asked to estimate the probability of developing rabies without rabies post-exposure prophylaxis for each indicated potential exposure scenario

^**b **^bite - superficial laceration, area not washed or cleaned; lick - over skin with recent superficial scratches, area not washed or cleaned; unk - unknown if true exposure, no obvious bites, scratches, or direct contact with bodily fluids

^**c **^Percentage of panel members recommending rabies post-exposure prophylaxis (PEP) for the indicated scenario

**Figure 1:**
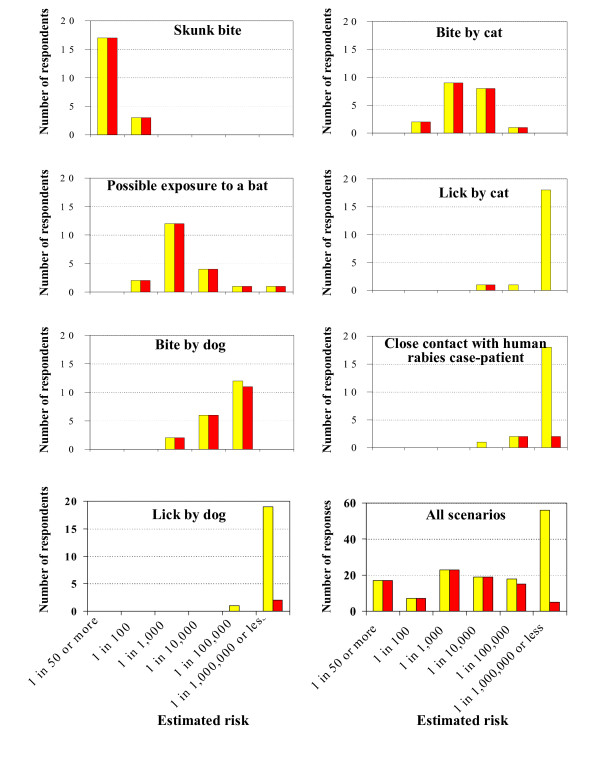
**Comparison of the estimated risk of rabies and PEP recommendations**. Respondents' estimates of the risk of transmission of human rabies without post-exposure prophylaxis (PEP) (shown in yellow) and the number recommending PEP (shown in red) in different potential exposure scenarios.

The panel judged that the second and fifth scenarios, which described a potential bat bite and superficial cat bite, respectively, to represent the next highest risk of rabies transmission, with a median risk of 0.001. However, these scenarios had wide variations. For example, for the bat scenario, estimates of the risk of transmission ranged from 0.01 to 0.000001, and the risk of transmission following a cat bite had an even wider range (Table 2). In both scenarios, all participants recommended PEP. The third scenario, which described a superficial dog bite, was graded as having a lower risk with a median value of 0.00001 (range: 0.001 to 0.00001). However, only one participant asserted that PEP was not necessary and the remainder noted that they would recommend rabies PEP. Comparison of the respondents' risk estimates versus their PEP recommendations are presented graphically in Figure 1.

For scenarios 4, 6, and 7, which describe a dog lick, a cat lick, and contact with a human rabies patient, respectively, over 90% of the participants estimated the risk to be <0.000001 (i.e. 1 in 1,000,000 or less). For these scenarios, panelists had differences during the initial rounds of the survey regarding rabies PEP recommendations. These differences resolved over subsequent rounds of the survey and over 90% of participants eventually did not recommend rabies PEP for scenarios 4 and 6. For scenario 7, 20% (4 of 20) participants recommended PEP, one participant was unsure, and the remainder recommended against administering rabies PEP.

## Discussion

Our panel estimated that the median risk of rabies transmission after bite exposure by a skunk, bat, cat, and dog was estimated to be 0.05, 0.001, 0.001, and 0.00001, respectively. All participants recommended rabies PEP in these scenarios. The median estimated risk after possible non-bite exposure to a dog, a cat, or human with rabies was less than 0.000001 and rabies PEP was usually not recommended. The estimates obtained in this analysis provide a key parameter for the economic analysis of public health measures for rabies prevention. In addition, these results provide information that may be useful in clinical decisions regarding the administration of rabies PEP in potential exposure scenarios.

Cases of human rabies are almost inevitably fatal. Thus, the decision to recommend for or against rabies PEP can be a life or death decision. When making a decision whether or not to recommend rabies PEP, the person making the recommendation must take into consideration widely differing risks based on the severity of exposure and the prevalence of rabies in the exposing animal species. Complicating the evaluation of the risks and benefits of administering rabies PEP is the fact that limited information may be available at the time a treatment decision must be made. The risk may be so small that is not possible to accurately characterize, leading to overreaction or over-evaluation of the risk, a common problem in many human health threats [14].

The complexity of rabies PEP decision-making is compounded by the all-or-none nature of the decision, the potential of treatment to reduce anxiety in the patient, and fear of litigation should rabies develop following the withholding of treatment. Furthermore, since either health insurance companies or state health departments invariably pay for rabies PEP, the patient's and/or health care provider's desire to reduce anxiety may not be aligned with the cost-effectiveness of rabies PEP treatment [6]. Thus, it is not surprising that decisions regarding when to administer PEP continue to challenge public health departments, emergency rooms, and primary care physicians.

From the estimates provided by the expert panel, it is possible to characterize the risk of zoonotic rabies transmission to humans following some non-bite exposures, as often being "negligible." For example, using the risk estimate of 1 in 1,000,000 following a lick from a cat or dog, and assuming there are 30,000 people who have this type of potential exposure each year in the United States, there would be only three cases of transmission per 100 years without ever giving rabies PEP in such instances. Conversely, the cost of administering rabies PEP to all persons with this type of exposure would be enormous. In New York State, from 1995-2000, nearly 30% of all rabies PEP treatment was given following "indirect" exposure [6]. Assuming that the assessments of risk in our study are reasonably correct, this statistic illustrates that there is significant potential to reduce the number of unnecessary rabies PEP given each year in the United States.

Our study is limited by the fact that the resultant estimates of risk are based solely on the opinions of experts. While these health professionals have extensive experience in assessing the potential risk of rabies transmission and the subsequent need for rabies PEP, purely objective data is currently unavailable. Thus, there remains a significant amount of uncertainty as seen in the relatively wide ranges of risk estimates provided by the panel. It is duly noted that the Delphi analysis method is inherently biased due to the composition and opinions of the participants. In order to reduce these biases, we included a large number of participants from various backgrounds who still had the relevant experience. In addition, all responses and discussion were managed anonymously so as to reduce individual predominance.

Since rabies prevalence and prevention practices vary greatly from state to state, our panel attempted to bridge this gap by including representatives from many different regions of the United States. While this may be useful in achieving a consensus estimate of risk, actual recommendations for rabies PEP should also take into account local animal species, surroundings, and prevalence rates.

## Conclusions

Estimating the risk of human rabies is a challenge for public health officials as the cost of rabies PEP is high and controlled studies in humans are neither feasible nor ethical. Our study provides important findings which have been incorporated into the Advisory Committee on Immunization Practices recommendations on the risk of rabies transmission to humans. The results may also provide guidance for more judicious use of rabies PEP in common potential exposure situations, including during a human rabies vaccine shortage [8]. Finally, these results provide information for human rabies prevention policy in the form of risk estimates for mathematical models of the cost-effectiveness of rabies PEP administration [15]. In an era of ever-increasing health care costs, innovative approaches are needed to identify scientifically valid and ethical methods of estimating the risk of human rabies transmission so that unnecessary vaccination can be avoided when it is not indicated.

## Abbreviations

PEP: Post-exposure prophylaxis

## Competing interests

The authors declare no competing interests. The findings and conclusions in this report are those of the author(s) and do not necessarily represent the views of the funding agency.

## Authors' contributions

SAV was the study coordinator, constructed the questionnaire, corresponded with all participants, analyzed the data, and drafted the manuscript. SEM and PD assisted with development of the questionnaire, data analysis, and drafting of the manuscript. MIM, CR, and HH participated in the design of the study and manuscript revision. DBF conceived of the study, participated in its design and coordination, and helped to draft the manuscript. All authors read and approved the final manuscript.

## Appendix 1

### Risk of Human Rabies Delphi Questionnaire

Approximate # of times you have made a professional recommendation regarding rabies PEP:

Approximate # of years of experience making professional recommendations regarding rabies PEP:

### Assumptions

• Estimated Human Rabies Mortality Rate from a Rabid Dog or Cat Without PEP [see Additional file 1]

• If the animal is caught but test results are not available within 48 h, the risk of human rabies and PEP administration will be considered equivalent to animal not available.

**Please answer the questions below to the best of your ability**.

1. An 11-year-old girl living in a state where **rabies is enzootic in skunks **is **bitten **by a **skunk **while playing in her backyard. She sustained a superficial laceration on her hand. The wound is not washed or cleaned. The animal cannot be found. Approximately 25% of skunks in the area have tested positive for rabies. The chance that the child will get rabies without rabies post-exposure prophylaxis is:

*Please indicate your choice with an X:*

A. 1 in 2 D. 1 in 20

B. 1 in 5 E. 1 in 100

C. 1 in 10 F. 1 in 1,000 or less

Do you recommend rabies post-exposure prophylaxis for this person?(Y/N)

Comments:

2. A mother living in a state where **rabies is enzootic in bats **states that she left her 9-month-old daughter sleeping in the next room. The child started crying and when the mother returned, she found a **bat **on the windowsill. The animal flew out the window and could not be found. Physical examination of the child showed ***no *obvious bites or skin abrasions**. Approximately 15% of bats in the area have tested positive for rabies. The chance that the infant will get rabies without rabies post-exposure prophylaxis is:

*Please indicate your choice with an X:*

A. 1 in 10 D. 1 in 10,000

B. 1 in 100 E. 1 in 100,000

C. 1 in 1,000 F. 1 in 1,000,000 or less

Do you recommend rabies post-exposure prophylaxis for this person?(Y/N)

Comments:

3. A 65-year-old woman living in a state where **rabies is enzootic in terrestrial animals **is **bitten **by an unprovoked stray **dog **while walking in the park. She sustained a superficial laceration on her hand. The wound is not washed or cleaned. The animal cannot be found. Approximately 0.1% of dogs in the area have tested positive for rabies. The chance that the woman will get rabies without rabies post-exposure prophylaxis is:

*Please indicate your choice with an X:*

A. 1 in 10 D. 1 in 10,000

B. 1 in 100 E. 1 in 100,000

C. 1 in 1,000 F. 1 in 1,000,000 or less

Do you recommend rabies post-exposure prophylaxis for this person?(Y/N)

Comments:

4. Same scenario as (3), except that the woman is only **licked **on the hand by the **dog**. The affected (licked) area has evidence of recent superficial scratches and is not washed or cleaned. The chance that the woman will get rabies without rabies post-exposure prophylaxis is:

*Please indicate your choice with an X:*

A. 1 in 10 D. 1 in 10,000

B. 1 in 100 E. 1 in 100,000

C. 1 in 1,000 F. 1 in 1,000,000 or less

5. A 15-year-old boy living in a state where **rabies is enzootic in raccoons **is **bitten **by an **unprovoked **stray **cat **while walking in a park. He sustained a superficial laceration on his hand. The wound is not washed or cleaned. The animal cannot be found. Approximately 1% of cats in the area have tested positive for rabies. The chance that the teenager will get rabies without rabies post-exposure prophylaxis is:

*Please indicate your choice with an X:*

A. 1 in 10 D. 1 in 10,000

B. 1 in 100 E. 1 in 100,000

C. 1 in 1,000 F. 1 in 1,000,000 or less

6. Same scenario as (5), except that the teenager is only **licked **on the hand by the **cat**. The affected (licked) area has evidence of recent superficial scratches and is not washed or cleaned. The chance that the teenager will get rabies without rabies post-exposure prophylaxis is:

*Please indicate your choice with an X:*

A. 1 in 10 D. 1 in 10,000

B. 1 in 100 E. 1 in 100,000

C. 1 in 1,000 F. 1 in 1,000,000 or less

7. A patient with unexplained encephalitis dies after a 10-day hospitalization. Rabies is diagnosed postmortem. You are asked by the nurse, who had close contact with the patient, if she needs rabies post-exposure prophylaxis. She did not wear gloves or a mask when caring for the patient. She denies having been bitten or scratched by the patient. The chance that the nurse will get rabies without rabies post-exposure prophylaxis is:

*Please indicate your choice with an X:*

A. 1 in 10 D. 1 in 10,000

B. 1 in 100 E. 1 in 100,000

C. 1 in 1,000 F. 1 in 1,000,000 or less

## Pre-publication history

The pre-publication history for this paper can be accessed here:

http://www.biomedcentral.com/1471-2458/10/278/prepub

## Supplementary Material

Additional file 1 **Table for Estimated Human Rabies Mortality Rate from a Rabid Dog or Cat Without PEP**. This was the information provided to Delphi participants adapted from Babes, B. (1912) *Traite de la Rage*. J.V. Bailliere, Paris, pp 81-119. Click here for file
