# Supplementary material for: Estimating the risk of rabies transmission to humans in the U.S.: a delphi analysis
Source: BMC Public Health. 2010 May 26;10:278. doi: 10.1186/1471-2458-10-278 (PMC2887820; doi:10.1186/1471-2458-10-278)
Supplement: Additional file 1 — Table for Estimated Human Rabies Mortality Rate from a Rabid Dog or Cat Without PEP. This was the information provided to Delphi participants adapted from Babes, B. (1912) Traite de la Rage. J.V. Bailliere, Paris, pp 81-119. [file 1471-2458-10-278-S1.DOC]

**Additional Files**

Additional File 1

| **Types of Exposure** | **Location** | **Estimated Mortality** |
| --- | --- | --- |
| Face | Multiple severe bites | 60-70% |
| Face | Single bite | 30-40% |
| Hand or fingers | Single bite | 15-20% |
| Hands | Superficial bite | 5% |
| Recent wound | Contact with rabid saliva | 0.1% |
| Wounds older than 24h | Contact with rabid saliva | 0.0% |
